# Efficient white phosphorescent organic light-emitting diodes using ultrathin emissive layers (<1 nm)

**DOI:** 10.1038/s41598-018-24434-8

**Published:** 2018-04-17

**Authors:** Haojian Yu, Xudong Dai, Fangnan Yao, Xiang Wei, Jin Cao, Chulgyu Jhun

**Affiliations:** 1 School of Materials Science and Engineering, Shanghai University, Shanghai, 200072 China; 2 Key Laboratory of Advanced Display and System Applications, Ministry of Education, Shanghai University, Shanghai, 200072 China; 3 School of Green Energy & Semiconductor Eng., Hoseo University, Asan City, Chungnam 336–795 South Korea

## Abstract

In this paper, efficient phosphorescent white organic light-emitting diodes (WOLEDs) were fabricated based on ultrathin doping-free emissive layers and mixed bipolar interlayers. The energy transfer processes were proved via the research of WOLEDs with different interlayer thicknesses and transient photoluminescence lifetime. WOLEDs with optimized thickness of doping-free emissive layers show maximum current efficiency of 47.8 cd/A and 44.9 cd/A for three-colors and four-colors WOLEDs, respectively. The Commission Internationale de L’Eclairage coordinates shows a very slight variation of ( ± 0.02, ± 0.02) from 5793 cd/m^2^ to 11370 cd/m^2^ for three-colors WOLEDs and from 3038 cd/m^2^ to 13720 cd/m^2^ for four-colors WOLEDs, respectively. The stability of the spectra is attributed to the stable and sequential energy transfer among the various dyes. The color temperature of four-colors WOLEDs can be obtained from 2659 to 6636 by adjusting the thickness of ultrathin emissive layer.

## Introduction

White organic light-emitting diodes (WOLEDs) have attracted much attention because of their advantages of low-cost, light weight, high-contrast, flexible and full-color^1–4^. Phosphorescent emitters can improve the efficiency of WOLEDs by harvesting both singlet and triplet energy for lighting, so it leads to a nearly 100% internal quantum efficiency (IQE) compared with fluorescent materials (25%)^5–7^.

To realize white-light emission covering the whole visible light, it is inevitable to utilize different dyes with diverse emissions. There are some general methods to fabricate WOLEDs, such as mixing multiple emitters into a single emission layer (EML)^8–10^, employing two or three EMLs structure with different emitters^11–15^, using stacking and tandem structure^16,17^. The traditional method to fabricate WOLEDs is co-evaporation the EMLs, which significantly improves electroluminescent (EL) efficiency^18^. However, it also brings some problems in entire fabrication process. It is difficult to precisely control the co-evaporation rate and dopant concentration, which leads to a bad reproducibility. Besides, the co-evaporation technology increases the cost, which is an adverse factor of commercialization. To solve these problems, there is a method to simplify the structures of devices by employing doping-free EMLs instead of the co-evaporation system. Since the first application of the doping-free WOLEDs (DF-WOLEDs) was demonstrated by Tsuji *et al*.^19^, a lot of attention have been paid^20–25^. Although the DF-WOLEDs have a great commercial potential because of the simple fabrication process, good reproducibility and low-cost, there are still some urgent problems need to be solved. For example, many studies were based on the research of devices using two kinds of emitters^26–28^. Lee *et al*.^20^ added yellow phosphorescent layer between the double blue phosphorescent emitters. Although the power efficiency is up to 35 lm/W at 1000 cd/m^2^, which is a comparable to that of the WOLEDs that are fabricated for achieving a yellow emission by using complicated host-guest doping system, two-colors WOLEDs are not suitable for lighting and display. On the other hand, Zhao *et al*.^25^ has fabricated three-colors and four-colors WOLEDs by using doping-free EMLs. They introduced ultrathin emission layers to the different position of hole transport layer (HTL) and electron transport layer (ETL) to realize the white emission. The power efficiencies were 29 lm/W and 19 lm/W at 1000 cd/m^2^ for the three-colors and four-colors WOLEDs, respectively. However, the power efficiency of WOLEDs based on the ultrathin doping-free EMLs is far from the requirement of practical lighting applications and lower than the traditional WOLEDs^29,30^, which is urgent to further improve. Besides, Liu *et al*.^31,32^ used blue fluorescent emission and phosphorescent emissions to fabricate hybrid WOLEDs which exhibit high-performance and long lifetime. Ma *et al*.^33,34^ doped phosphorescent materials into bipolar-transporting host to reduce the exciton quenching. However, there is little research on doping-free phosphorescent EMLs with bipolar interlayers.

In this paper, bipolar interlayers and ultrathin doping-free phosphorescent EMLs were adopted. The hole-transporting material 4,40,400-tri(N-carbazolyl) triphenylamine (TCTA) and electron-transporting materials 1,3,5-tri(m-pyrid-3-yl-phenyl) benzene (Tm_3_PyPB) were mixed as the bipolar interlayers. According to our previous study, the current density of hole only devices and electron only devices were close when the mass ratio of TCTA: Tm_3_PyPB was 3.5: 1^35^. The thicknesses of bipolar interlayers were changed to adjust the energy transfer from short-wave emitters to long-wave emitters. Moreover, the working mechanism of the phosphorescent WOLEDs was discussed, and we concluded that the combined effects of stable energy transfer and even excitons distribution were the factor for achieving the relatively stable spectra. As a result, the high efficiency phosphorescent WOLEDs can be achieved by use of doping-free phosphorescent EMLs and mixed bipolar interlayers.

## Methods

### Materials

**Figure 1:**
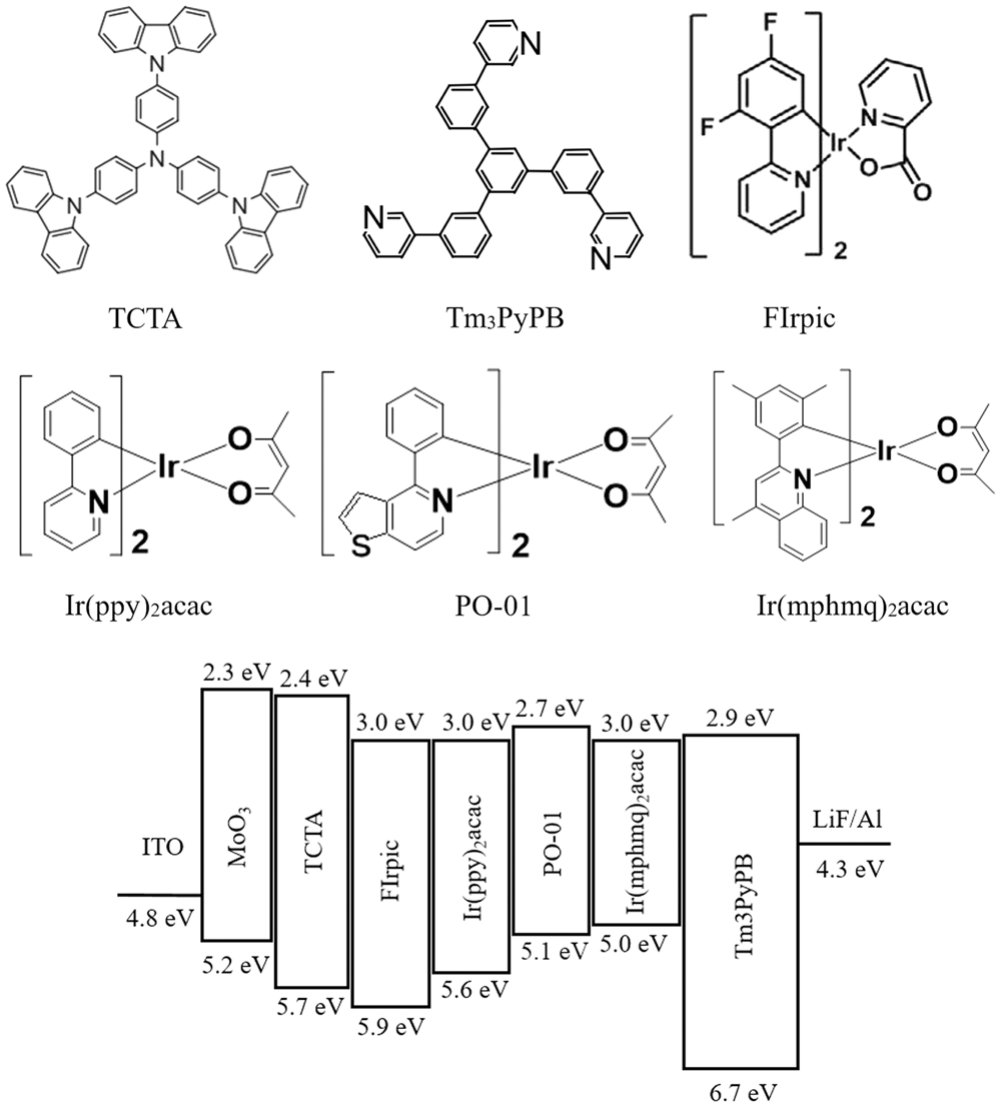
Chemical structures of materials and detailed energy level diagram.

All compounds were purchased from commercial sources unless noted and used without further purification. Figure 1 shows the chemical structures and detailed energy level diagram of the materials proposed. The highest occupied molecular orbital (HOMO) level of MoO_3_ (HOMO = 5.2 eV) is between ITO (HOMO = 4.8 eV) and TCTA (HOMO = 5.7 eV), so MoO_3_ is beneficial to the hole injection^36^. Meanwhile, 1 nm LiF is used to enhance the electrons injection. Since TCTA has a relatively poor electron transport ability, which merely has an electron-mobility of 10^−8^ cm^2^/Vs^37^, and the electron-injection barrier between the lowest unoccupied molecular orbitals (LUMO) of Tm_3_PyPb (LUMO = 2.9 eV) and TCTA (LUMO = 2.40 eV) is merely 0.5 eV, it is difficult for electrons to be injected from Tm_3_PyPb into TCTA. Therefore, TCTA and Tm_3_PyPb can be a good confinement zone for excitons. Because the fact that the triplet energy levels of TCTA (2.86 eV) and Tm_3_PyPB (2.8 eV) are higher than that of all four phosphorescent dyes, the excitons generated between TCTA and Tm_3_PyPb can effectively transfer to dyes and then generate efficient emission.

### Device fabrication

Various layers of WOLEDs were fabricated by a vacuum evaporation technique. The thickness value and deposition rates of the materials were monitored by an oscillating quartz thickness monitor. All layers were fabricated on a glass substrate with a patterned indium tin oxide (ITO), which is 150 nm thickness, ~10 Ω/sq. The ITO glasses were cleaned for 15 min in a sequential ultrasonic bath with detergent, de-ionized water, acetone and isopropanol, and then cleaned in a UV-ozone chamber for 15 min. The HTL, electron blocking layer (EBL), EML, ETL, electron injection layer (EIL) and the cathode (Al) were deposited by thermal evaporation at a high vacuum of ~10^-4^ Pa. The MoO_3_ doped TCTA as a p-type HTL was co-evaporated onto the ITO substrate at the speeds of 0.4 Å/s and 0.6 Å/s, respectively. Then TCTA was evaporated as EBL on the top of HTL layer. FIrpic, Ir(ppy)_2_acac, PO-01 and Ir(mphmq)_2_acac were evaporated as dyes at a speed of 0.1 Å/s. The mixed interlayers were evaporated among different dyes at a speed of 1 Å/s. Dyes and the mixed interlayers were combined as EML. A 40-nm-thick Tm_3_PyPB was deposited as ETL at a rate of 1 Å/s following the EML. Finally, a ~1-nm-thick LiF and ~120-nm-thick Al cathode were sequentially deposited. The deposition rates of LiF and Al were 0.1 and 5.0 Å/s, respectively. The active areas were 3 × 3 mm^2^ in all devices.

### Measurements

The voltage-current density, luminance and EL spectra were measured with a programmable source meter (Keithley 2400), luminance meter (LS110, Konica Minolta) and a spectrophotometer (Spectrascan PR670, Photo Research). The photoluminescence (PL) spectra and transient PL lifetime were measured by FLSP 920 spectrometer series.

## Result and Discussion

### Two-colors OLEDs devices research

In order to verify the characteristic of energy transfer in the OLEDs device, the thickness of interlayer between blue and green dyes was investigated. Devices with four different thickness of interlayers were proposed. The structure of the devices was ITO/MoO_3_: TCTA (2:3, 35 nm)/TCTA (10 nm)/TCTA: Tm_3_PyPB (3.5:1, 20-X nm)/Ir(ppy)_2_acac (0.15 nm)/TCTA: Tm_3_PyPB (3.5:1, X nm)/FIrpic (0.4 nm)/Tm_3_PyPB (40 nm)/LiF (1 nm)/Al (120 nm), where X = 0,2,3,4.

**Figure 2:**
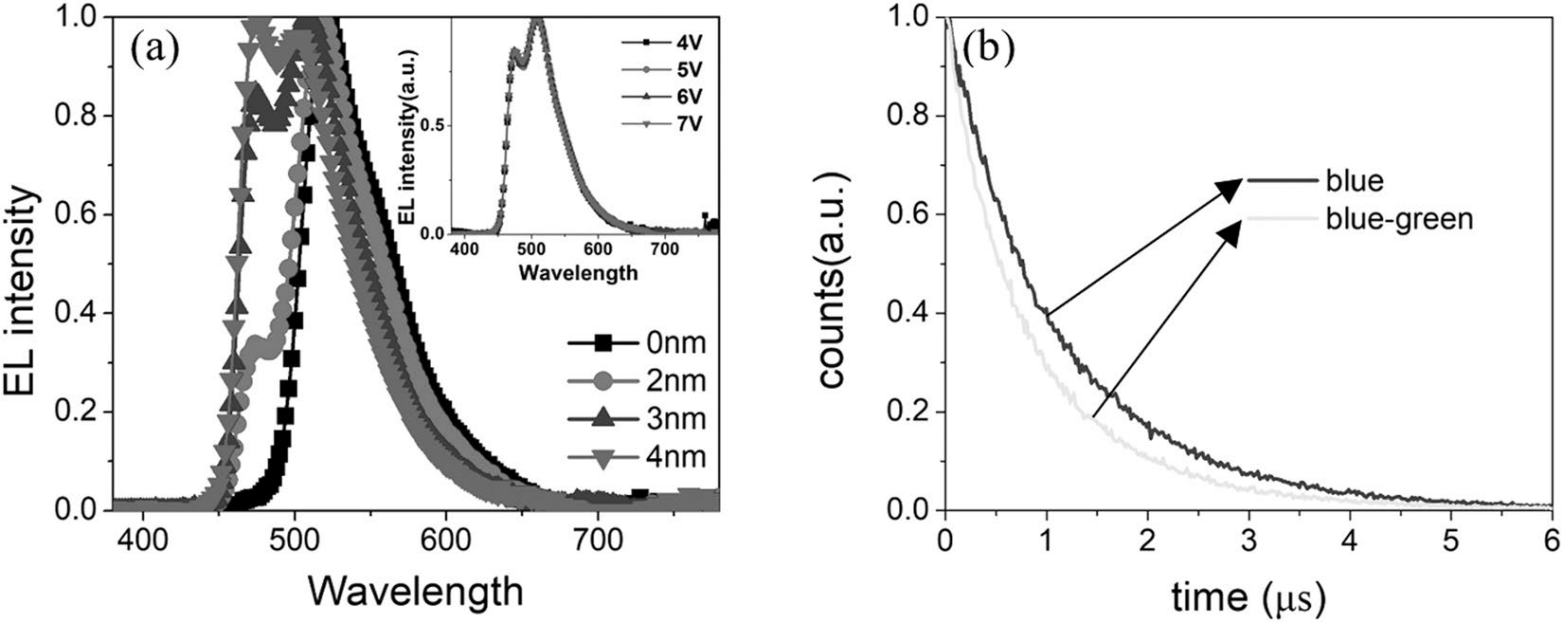
(**a**) EL spectra of the devices with different thickness of interlayers that between blue and green dyes at 6 V. Inset: the EL spectra of the device with 3 nm interlayer at different operating voltages. (**b**) Transient PL lifetime of blue film and blue-green film

Figure 2(a) shows the normalized EL spectra of the four devices at 6 V. When there is no interlayer between blue and green dyes, the device only shows a main green emission from Ir(ppy)_2_acac. With the increase of interlayer thickness, the blue emission appeared and gradually enhanced. So we can conclude that the energy transfer from blue to green dye can be controlled by the thickness of interlayer. The spectra of devices achieved an optimal balance when the thickness of interlayer is 3 nm. We can see that the spectra show a good stability due to the stable and sequential energy transfer from blue dye to green dye. The Commission Internationale de L’Eclairage (CIE) coordinates of the device with 3 nm interlayer are (0.209, 0.460), (0.209, 0.459), (0.207, 0.458) and (0.206, 0.455) at the operating voltage of 4 V, 5 V, 6 V and 7 V (which correspond to the luminance of 548 cd/m^2^, 2446 cd/m^2^, 5438 cd/m^2^ and 7996 cd/m^2^), respectively. The EL performances of devices with different thickness of interlayer between blue and green dyes are shown in Fig. S1, Supporting Information. Considering the triplet energy lever of blue dye is higher than green dye, energy transfer between blue and green dye is reasonable. It has been reported that the exciton lifetime will change with the advent of energy transfer^38,39^. If the energy transfer from the donor to the acceptor happens, the lifetime of the donor will decrease. The mixed bipolar material (TCTA: Tm_3_PyPB = 3.5: 1) was utilized as co-host, and then emitting dyes were doped into the co-host. Finally, the energy transfer can be confirmed by measuring the transient PL lifetime of the emitting dyes. Blue dye was separately doped into co-host, as depicted in Fig. 2(b), and the lifetime of blue emission was fitted to be about 1.21 μs (τ1) at 470 nm. When blue and green dye were doped into co-host together, the lifetime of blue emission dropped to 0.98 μs (τ_2_). According to the equation^40^

1 $${{\rm{\eta }}}_{{\rm{ET}}}=1-{{\rm{\tau }}}_{2}/{{\rm{\tau }}}_{1}$$

where η_ET_ is the energy transfer ratio from the donor to the acceptor, η_ET_ is 19.0%. It proves that there indeed exists an energy transfer process from FIrpic to Ir(ppy)_2_acac.

### Three-colors WOLEDs devices research

On the basis of two-colors devices, orange dye was added to achieve three-colors warm WOLEDs. The structure of the three-colors devices is ITO/MoO_3_: TCTA (2:3, 35 nm)/TCTA (30-Y nm)/PO-01 (0.4 nm)/TCTA: Tm_3_PyPB (3.5:1, Y nm)/Ir(ppy)_2_acac (0.15 nm)/TCTA: Tm_3_PyPB (3.5:1, 3 nm)/FIrpic (0.1 nm)/Tm_3_PyPB (40 nm)/LiF (1 nm)/Al (120 nm). Here, Y is 3,4 and 5 corresponding to devices T1, T2 and T3.

**Figure 3:**
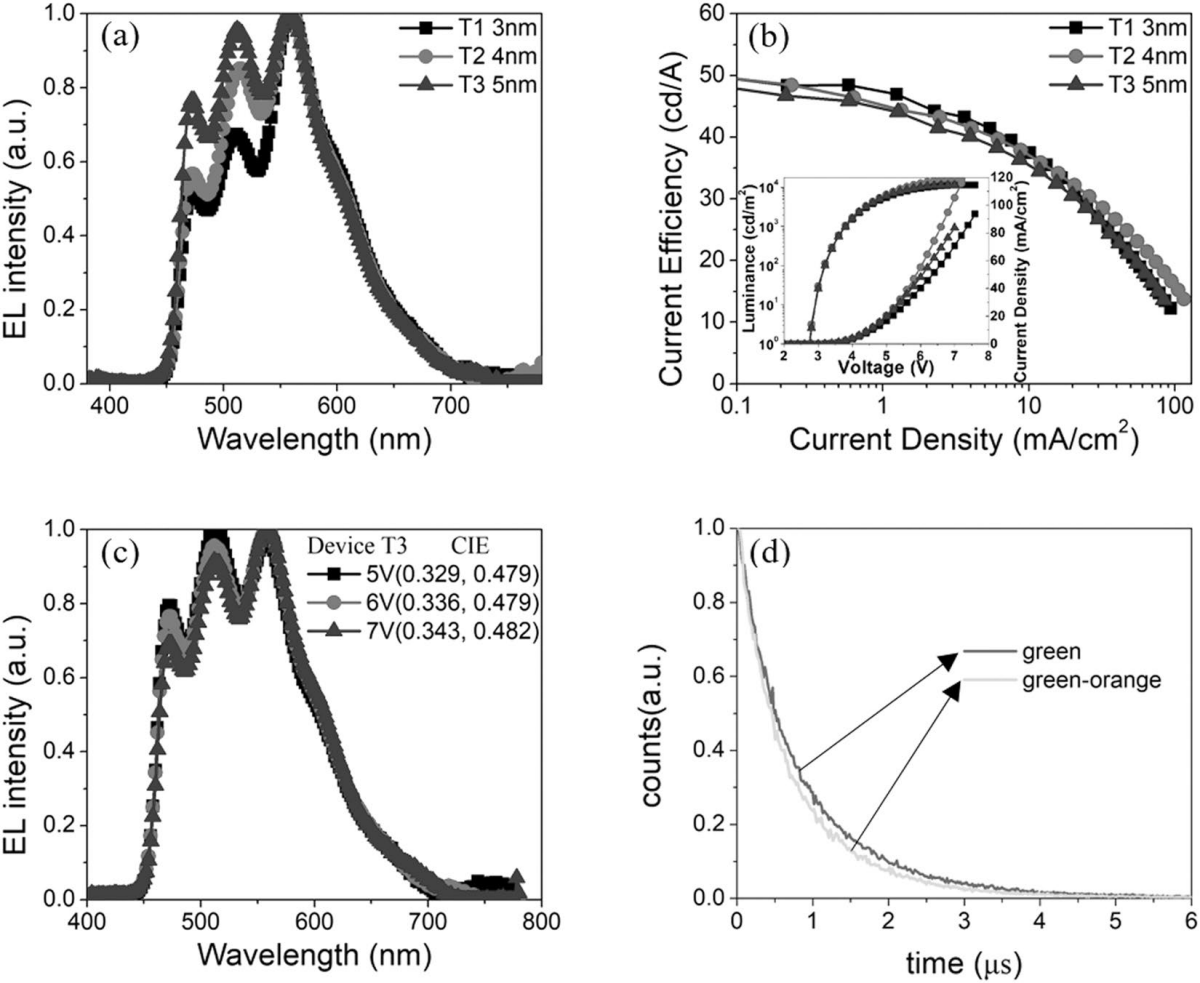
(**a**) EL spectra of devices (T1, T2 and T3) with different thickness of interlayers that between green and orange dyes at 6 V. **(b**) Current efficiencies versus current density of devices T1, T2 and T3. Inset: Current density-Voltage-Luminescence J-V-L characteristic of devices T1, T2 and T3. (**c**) The EL spectra of the device T3 at different operating voltages. (**d**) Transient PL lifetime of green film and green-orange film.

The EL spectra of devices T1, T2 and T3 are normalized to the orange emission peak and shown in Fig. 3(a). The orange emission peak is much higher than green emission peak in the device T1 with 3 nm interlayer between PO-01 and Ir(ppy)_2_(acac). It illustrates that the energy transfer from Ir(ppy)_2_(acac) to PO-01 is too strange. So the thickness of interlayer between PO-01 and Ir(ppy)_2_(acac) was added. A better spectrum appeared When the interlayer reached 5 nm, the orange emission peak was almost equal with green emission peak. Although the current efficiency is dropped with the weakening of the orange emission, the efficiencies of device T3 is still comparable with device T1 and T2. As shown in Fig. 3(b), the maximum current efficiencies of device T1, T2 and T3 are 49.5 cd/A, 49.6 cd/A and 47.8 cd/A, respectively. The maximum power efficiencies and external quantum efficiencies (EQE) of the three devices are 50.5 lm/W (16.0%), 50.7 lm/W (16.1%) and 48.8 lm/W (15.4%) accordingly as shown in Fig. S2(a) and S2(b), Supporting Information. All the devices show the turn-on voltages is about 2.73 V ( ± 0.1 V), which is as low as the triplet energy gap (triplet energy~2.7 eV) of phosphorescent dye, FIrpic. The detailed EL datum of devices T1~T3 is listed in the Table 1. We can see the spectra just show a little change in Fig. 3(c). The CIE coordinates of the device T3 are (0.329, 0.479), (0.336, 0.479) and (0.343, 0.482) at the operating voltage of 5 V, 6 V and 7 V (which correspond to the luminance of 5793 cd/m^2^, 10240 cd/m^2^ and 11370 cd/m^2^), respectively. The color-rendering index (CRI) of device T3 is 54.2 at 7 V. Green dye was separately doped into co-host, as depicted in Fig. 3(d), and the lifetime of green emission was fitted to be about 0.95 μs at 520 nm. The lifetime of green emission dropped to 0.82 μs while green and orange dyes were doped into co-host together, and the energy transfer ratio is η_ET_ = 1 – 0.82/0.95 = 13.9%. The stable and sequential energy transfer also exists between Ir(ppy)_2_(acac) and PO-01. Meanwhile, since interlayer was bipolar materials, both holes and electrons can flexibly transport across it, resulting in the relatively even excitons distribution. As shown in Fig. S2(c), the spectrum exhibits three main emission peaks from the PO-01, Ir(ppy)_2_(acac) and FIrpic at low operating voltage of 3.5 V (which correspond to the luminance of 365 cd/m^2^). A relatively weak orange emission can be ascribed to the fact that more triplet excitons created near cathode side (blue dye side) at low voltages since the doped MoO_3_ enhanced the hole injection/transport^36^. With the increase of voltage, more excitons distributed near orange dye side (anode side), which leaded to the enhancement of orange emission. Under the combined effect of stable energy transfer and even excitons distribution at high voltage (above 5 V), relatively stable spectra were obtained from 5793 cd/m^2^ to 11370 cd/m^2^.

**Table 1:** The detailed EL performances of three-colors WOLEDs.

Device	V_on_(V)^a^	CE (cd/A)		PE (lm/W)		EQE (%)	
		Max^b^	100^c^	Max^b^	100^c^	Max^b^	100^c^
**T1**	2.72	49.5	48.7	50.5	47.5	16.0	15.8
**T2**	2.73	49.6	48.4	50.7	47.6	16.1	15.9
**T3**	2.74	47.8	46.7	48.8	45.9	15.4	15.1

^a^At 1 cd/m^2^.

^b^The maximum efficiency.

^c^The efficiency at the luminance of 100 cd/m^2^.

Besides, the devices that used TCTA or Tm_3_PyPB as interlayers were fabricated. However, the TCTA and Tm_3_PyPB cannot play a good role in energy transfer alone and only one or two main emission can be saw.

### Four-colors WOLEDs devices research

**Figure 4:**
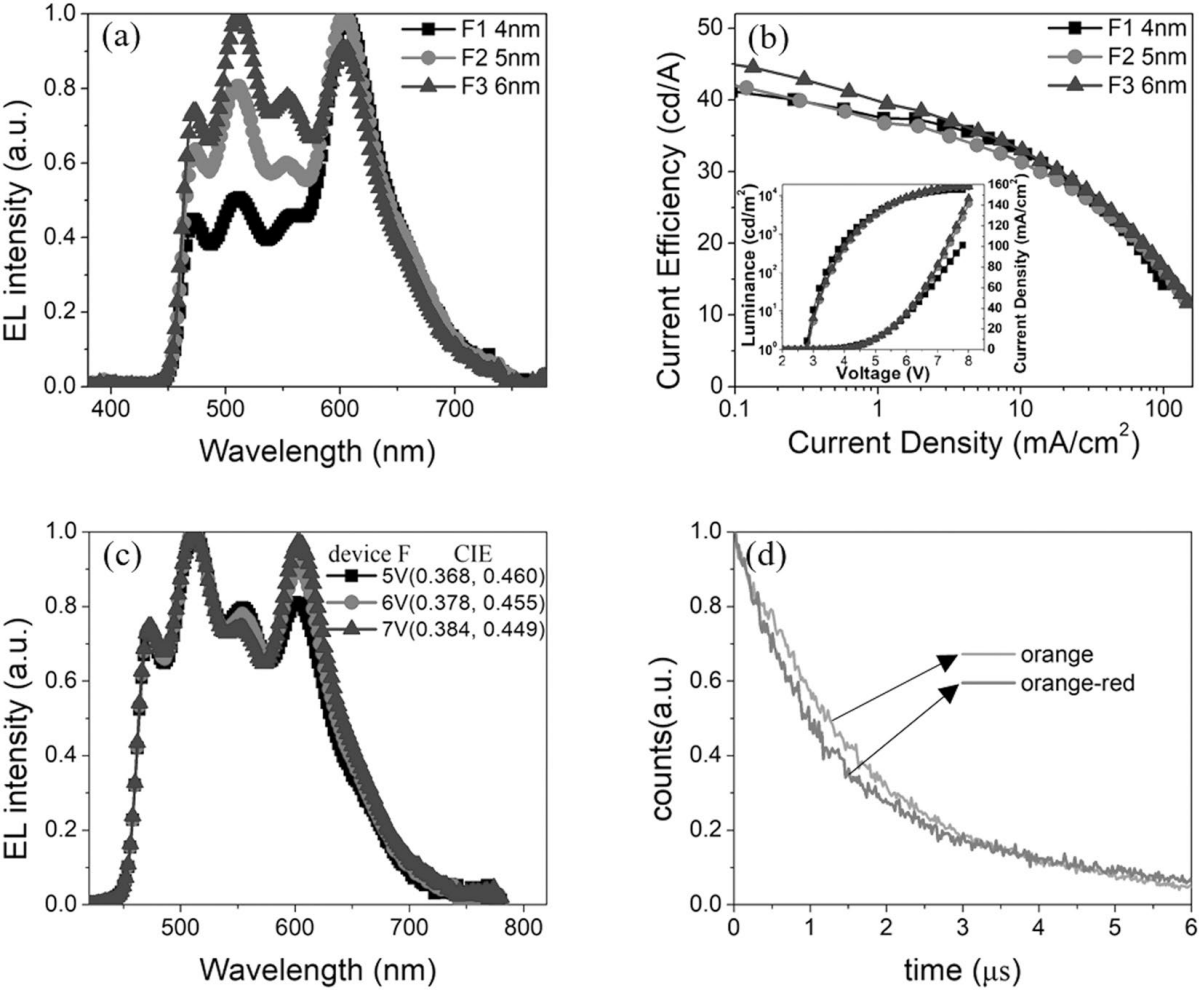
(**a**) EL spectra of devices (F1, F2 and F3) with different thickness of interlayers that between orange and red dyes at 6 V. (**b**) Current efficiencies versus current density of devices F1, F2 and F3. Inset: Current density-Voltage-Luminescence J-V-L characteristic of devices F1, F2 and F3. (**c**) The EL spectra of the device F3 at different operating voltages. (**d**) Transient PL lifetime of orange film and orange-red film.

To achieve four-colors WOLEDs, red dye was added on the basis of three colors devices. The structure of the four-colors devices is ITO/MoO_3_: TCTA (2:3, 35 nm)/TCTA (27-Z nm)/Ir(mphmq)_2_acac (0.07 nm)/TCTA: Tm_3_PyPB (3.5:1, Z nm)/PO-01 (0.2 nm)/TCTA: Tm_3_PyPB (3.5:1, 3 nm)/Ir(ppy)_2_acac (0.15 nm)/TCTA: Tm_3_PyPB (3.5:1, 3 nm)/FIrpic (0.1 nm)/Tm_3_PyPB (40 nm)/LiF (1 nm)/Al (120 nm). Here, Z is 4, 5 and 6 corresponding to devices F1, F2 and F3. When the four-colors device F1 was fabricated, we find that the intensity of Ir(mphmq)_2_acac was too strong. So the thickness of interlayer between Ir(mphmq)_2_(acac) and PO-01 was added. As shown in Fig. 4(a), the thicker the interlayer is, the weaker the red emission is. As we can see, a good white spectrum was obtained when thickness of interlayer between Ir(mphmq)_2_acac and PO-01 was 6 nm. Figure 4(b) shows the current efficiencies of devices F1, F2 and F3. With the reduction of red emission, the current efficiency improved. The maximum current efficiencies of device F1, F2 and F3 are 41.2 cd/A, 41.9 cd/A and 44.9 cd/A, respectively. As shown in Fig. 4(b) inset, the turn-on voltages of device F1, F2 and F3 are 2.77 V, 2.81 V and 2.80 V, respectively. The maximum power efficiencies and EQE of the three devices are 40.3 lm/W (16.9%), 39.3 lm/W (16.0%) and 42.5 lm/W (16.1%) accordingly as shown in Fig. S3(a) and S3(b), Supporting Information. The spectrum of the device F3 is shown in Fig. 4(c) and CIE coordinates of the device F3 are (0.368, 0.460), (0.378, 0.455) and (0.384, 0.449) at the operating voltage of 5 V, 6 V and 7 V (which correspond to the luminance of 3038 cd/m^2^, 8314 cd/m^2^ and 13720 cd/m^2^), respectively. The CRI of device F3 is 77.4 at 7 V. As depicted in Fig. 4(d), the lifetime of orange emission was fitted to be about 1.80 μs at 550 nm. The lifetime of green emission dropped to 1.38 μs When orange and red dyes were doped into co-host together. The energy transfer ratio from PO-01 to Ir(mphmq)_2_acac is η_ET_ = 1 – 1.38/1.80 = 23.3%. It illustrates that there is energy transfer between PO-01 and Ir(mphmq)_2_acac. Meanwhile, as shown in Fig. S3(c), there were more triplet excitons near cathode side (blue dye side) due to the adoption of MoO_3_, which lead to the red emission (anode side) was relatively weak at the low operating voltage of 4 V (which correspond to the low luminance of 424 cd/m^2^). With the increase of voltage, the enhancement of red emission can be ascribed to the fact that more excitons distributed near red dye side (anode side). Under the combined effect of stable energy transfer and even excitons distribution at high voltage (above 5 V), relatively stable spectra were obtained from 3038 cd/m^2^ to 13720 cd/m^2^.

Since there is energy transfer between yellow and red dyes, changing the thickness of PO-01 can affect not only the intensity of orange light, but also the intensity of red light. The thickness of PO-01 in device F3 (the thickness of PO-01 was 0.2 nm) was changed, and devices F4 (the thickness of PO-01 was 0.1 nm) and F5 (the thickness of PO-01 was 0.3 nm) were fabricated.

**Figure 5:**
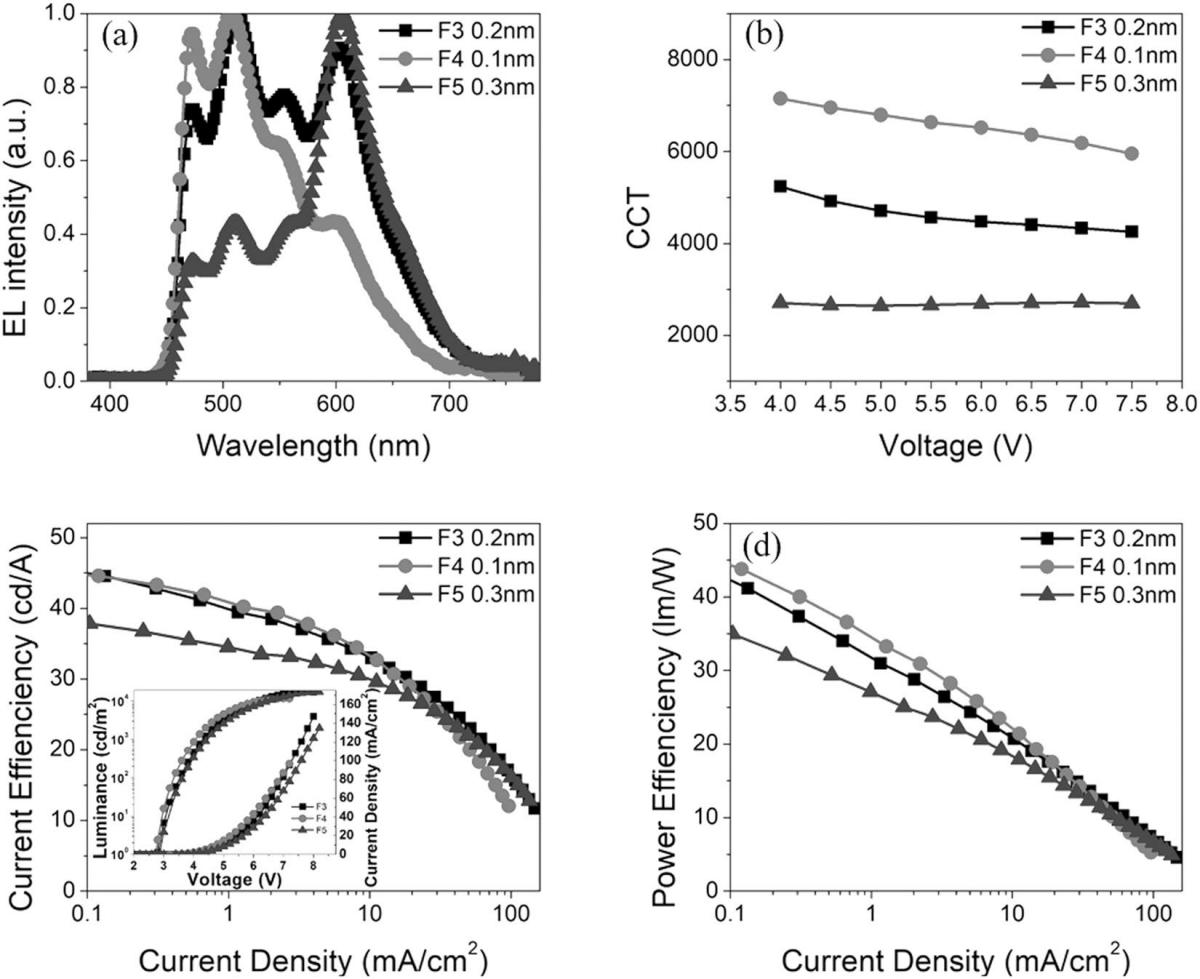
EL performances of the devices with different thickness of orange dyes. (**a**) EL spectra of devices F3, F4 and F5 at 6 V. (**b)** CCT of devices F3, F4 and F5 at 6 V. (**c**) Current efficiencies versus current density of devices F3, F4 and F5. Inset: Current density-Voltage-Luminescence J-V-L characteristic of devices F3, F4 and F5. (**d**) Power efficiencies versus current density of devices F3, F4 and F5.

Figure 5(a) depicts the EL spectra of devices F3, F4 and F5 at 6 V. We can see that the thicker the PO-01 layer is, the stronger the red emission is. In other word, thickening the PO-01 layer can increase the orange-to-red energy transfer. The color correlated temperature (CCT) of devices F4, F3 and F5 are 6636, 4565 and 2659 at 5000 cd/m^2^, respectively. Since the CCT can be adjusted by changing the thickness of PO-01, we can get a sun-like emission covering the entire daylight at different times (i. e., 2500, 3500 and 5000 K at sunset, sunrise and noon, respectively). Figure 5(c) and (d) shows the efficiencies of devices F3, F4 and F5. The maximum efficiencies of device F4 and F5 are 44.9 cd/A (44.5 lm/W) and 37.7 cd/A (35.1 lm/W), respectively. As shown in Fig. S3(d), the maximum EQE of the device F4 and F5 are15.3% and 16.4%, respectively. As shown in Fig. 5(c) inset, the turn-on voltages of the device F4 and F5 are 2.75 V and 2.85 V, respectively. The detailed EL datum of devices F1~F5 are listed in the Table 2.

**Table 2:** he detailed EL performances of four-colors WOLEDs.

Device	V_on_(V)^a^	CE (cd/A)		PE (lm/W)		EQE (%)		CCT
		Max^b^	100^c^	Max^b^	100^c^	Max^b^	100^c^	5000^d^
F1	2.77	41.2	40.1	40.3	37.0	16.9	16.5	3014
F2	2.81	41.9	39.9	39.3	34.8	16.0	15.3	3865
F3	2.80	44.9	42.8	42.5	37.4	16.1	15.4	4565
F4	2.75	44.9	43.3	44.5	40.0	15.3	14.7	6636
F5	2.85	37.7	36.7	35.1	32.1	16.4	15.9	2659

^a^At 1 cd/m^2^.

^b^The maximum efficiency.

^c^The efficiency at the luminance of 100 cd/m^2^.

^d^Color correlated temperature at the luminance of 5000 cd/m^2^.

## Conclusions

In summary, we reported an efficient WOLEDs based on ultrathin doping-free EMLs and mixed bipolar interlayers, and verified the existence of energy transfer among various dyes. The three-colors and four-colors WOLEDs have maximum efficiencies of 47.8 cd/A (48.8 lm/W) and 44.9 cd/A (42.5 lm/W), respectively, while the maximum EQE of the two devices are15.4% and 16.1% accordingly. The CIE coordinates of the three-colors and four-colors WOLEDs show a very slight variation. A sun-like emission covering the CCT of entire daylight at different times has been obtained by changing the thickness of PO-01. Besides, phosphorescent OLEDs usually show poor lifetime, which has been attributed to the traps caused by bimolecular triplet-polaron annihilation (TPA)^41^. In our devices, the adoption of ultrathin phosphorescent EML (<1 nm) and bipolar interlayers can broaden the exciton distribution zone and decrease the exciton density in EMLs. Therefore, the TPA will be reduced^41^, and it is hopeful to improve the OLEDs lifetime.

## Electronic supplementary material

Supplementary Information
