# Supplementary material for: Efficient white phosphorescent organic light-emitting diodes using ultrathin emissive layers (<1 nm)
Source: Sci Rep. 2018 Apr 17;8:6068. doi: 10.1038/s41598-018-24434-8 (PMC5904154; doi:10.1038/s41598-018-24434-8)
Supplement: Supplementary file 1 — Supplementary Information [file 41598_2018_24434_MOESM1_ESM.docx]

**Supporting Information**

**Efficient white phosphorescent organic light-emitting diodes using ultrathin emissive layers（<1 nm）**

Haojian Yu^1^, Xudong Dai^1^, Fangnan Yao^1^, Xiang Wei^1^, Jin Cao^2^*, Chulgyu Jhun^3^

**Corresponding authod: cj2007@shu.edu.cn.*

*^1^School of Materials Science and Engineering, Shanghai University, Shanghai 200072, China.*

*^2^Key Laboratory of Advanced Display and System Applications, Ministry of Education, Shanghai University, Shanghai 200072, China.*

*^3^School of Green Energy & Semiconductor Eng., Hoseo University, Asan City, Chungnam, 336-795, South Korea*

**Fig. S1** EL performances of devices with different thickness of interlayers that between blue and green dyes. (a) Current efficiencies versus current density of the devices. (b) EQE versus current density of the devices. (c) Power efficiencies versus current density of the devices. (d) Current density-Voltage-Luminescence J-V-L characteristic of the devices.

**Fig. S2** (a) EQE versus current density of devices (T1, T2 and T3) with different thickness of interlayers that between green and orange dyes. (b) Power efficiencies versus current density of devices T1, T2 and T3. (c) The EL spectra of the device T3 at different operating voltages.

**Fig. S3** (a) EQE versus current density of devices (F1, F2 and F3) with different thickness of interlayers that between orange and red dyes. (b) Power efficiencies versus current density of devices F1, F2 and F3. (c) The EL spectra of the device F3 at different operating voltages. (d) EQE versus current density of devices (F3, F4 and F5) with different thickness of orange dyes.
